# Investigation of the influencing factors with the uptake of the COVID‐19 vaccine booster dose among the general population of Ardabil, Iran: A cross‐sectional study

**DOI:** 10.1002/hsr2.1494

**Published:** 2023-08-22

**Authors:** Nazila NeJhaddadgar, Mohammad Jafarzadeh, Zahra Khazir, Javad Yoosefi Lebni, Mohammad Rostami, Parisa Janjani, Arash Ziapour

**Affiliations:** ^1^ Social Determinants of Health Research Center Ardabil University of Medical Sciences Ardabil Iran; ^2^ Department of Infectious Diseases, School of Medicine Ardabil University of Medical Sciences Ardabil Iran; ^3^ Tabas School of Nursing Birjand University of Medical Sciences Birjand Iran; ^4^ Social Determinants of Health Research Center Lorestan University of Medical Sciences Khorramabad Iran; ^5^ Students Research Committee Paramedical School of Kermanshah University of Medical Sciences Kermanshah Iran; ^6^ Cardiovascular Research Center, Health Institute, Imam‐Ali Hospital Kermanshah University of Medical Sciences Kermanshah Iran

**Keywords:** attitudes, COVID‐19, periodically dose, perspectives, vaccination, vaccine booster

## Abstract

**Background and Aims:**

Vaccination is one of the most efficient approaches to combating COVID‐19 if it is adequately embraced by the general population. Numerous factors influence the uptake or refusal of the booster dose. The goal of this study was to look at the different factors that affect how the general population in Ardabil feels about getting vaccine boosters (annual boosters) for COVID‐19 and to evaluate those feelings.

**Methods and Materials:**

In the city of Ardabil, general population, perceptions towards the COVID‐19 vaccine booster (annual boosters) dose were evaluated using a cross‐sectional survey design between January 2 and March 25, 2022. A questionnaire was developed and filled out by 662 subjects via phone calls from healthcare providers. Descriptive statistics, the Chi‐square test, the correlation coefficient, and regression analysis were run for the analysis of quantitative data.

**Results:**

The findings of the research revealed that 238 participants, or 35.9%, had previously gotten the booster dose of the COVID‐19 vaccination, while 198 participants, or 29.2%, expressed a desire to do so as soon as feasible. A total of 187 (28.2%) respondents reported not wanting to get a booster dose, and 39 (5.7%) could not decide. In the factors found to affect decisions not to accept regular doses, adverse effects (45.4%) and the presence of misinformation (30%) were the most important. Regression in educational achievement, and following the COVID‐19 news showed to be the major predictors of the subjects' attitudes toward the regular COVID‐19 vaccine.

**Conclusion:**

The present findings revealed that low confidence in the efficiency of the booster shot and misinformation are two critical factors to consider in educational planning and interventions.

## 1 INTRODUCTION

The coronavirus disease was originally caused by the SARS‐CoV‐2 virus, also known as COVID‐19, which is an invasive condition. This infection was found for the first time in Wuhan, which is located in the Hubei region of China. From there, it moved on to other nations.^1^, ^2^ After a follow‐up period of almost 2 years, it has become clear that adequate access to effective vaccinations and the adherence of health standards are essential to putting a stop to the COVID‐19 outbreak and lowering the risk of severe illness and hospitalization.^3^, ^4^, ^5^, ^6^, ^7^, ^8^ The COVID‐19 vaccines have proved efficient in dealing with major diseases, hospital stay, and mortality caused by affliction with various SARS‐CoV‐2strains. Antibody levels were used by the producers of COVID‐19 vaccines as surrogate biomarkers to reveal the efficacy of vaccine. In fact, not unlike many vaccinations, the antibody levels of COVID‐19 slowly decrease after vaccinations.^9^, ^10^, ^11^, ^12^, ^13^

According to findings of a progressive reduction in illness after using the vaccine and the inhibitory role of the booster dose 6 months after the main vaccination, several more countries have agreed to administer a booster shot to people according to age categories.^14^, ^15^, ^16^, ^17^ Also The booster dose of the vaccine increases the antibody titer and reduces the chance of a widespread epidemic, and as a result, the number of severe patients and mortality decreases.^15^, ^18^ This decision was made based on the testimony of the steady reduction in contagion after using the vaccine.^8^, ^14^, ^15^, ^16^ Because it only takes a few months for vaccination proficiency to fall from 74.7% to 53.1% in a survey of fully vaccinated people^2^ several countries have begun administering booster doses by injection.^19^, ^20^ In the fight against the pandemic, the administration of booster injections will, at some point, prove to be a very important tactic. The majority of the authorities working in the various health care systems are concerned about how well a booster dosage will be received.^20^ Overall, there is a consensus on the fat that those who got two doses of COVID‐19 vaccines need to get later doses periodically.^21^ These further doses have proved to be useful and safe.^2^ In light of the fact that COVID‐19 immunization attempts have been thwarted by false information,^22^, ^23^, ^24^, ^25^ it is expected that COVID‐19 booster shot programs will meet similar obstacles. Studies of the reception of COVID‐19 vaccine booster doses are scarce. A study showed that about 50% of qualified people were concerned about vaccination side effects that prevented them from receiving a booster dose, and 45.3% reckoned that getting a third dose of the vaccine could worsen the adverse effects. Concerns about getting vaccinated are not surprising. As an instance, the polio vaccination plan in Pakistan became troublesome due to public concern about the quality of the vaccine. Another instance of vaccine hesitancy happened during an epidemic of influenza in America to convince pregnant women to get the vaccine.^24^ Given that the decision to get a booster dose of the COVID‐19 vaccine is the result of a complex interaction between different variables, it is hard to think of an unambiguousimage of potential perceptions of vaccination in the overall public. The present research explored factors that influence the uptake of the COVID‐19 vaccine booster dose in the overall population of Ardabil (a historical city in the northwestern part of Iran, which is the capital of Ardabil Province).

## 2 MATERIALS AND METHODS

### 2.1 Design and setting of study

Between January 2 and March 25, 2022, research was done using a cross‐sectional design. Six hundred sixty‐two people in the city of Ardabil, ranging in age from 18 to 80 years, were asked to participate in this study. Using a web‐based survey, the purpose of this study was an investigation of the influencing factors towards the uptake of the COVID‐19 vaccine booster dose in the general public. Due to the fact that the government had ordered a lockdown, it was impossible to conduct a community‐based nationwide sample poll. The sample size was estimated at 662 with a 95% confidence interval (CI), 0.5% margin of error, and a 25% expected agreement. The website Survey Monkey and the recruitment of participants relied on convenience (non‐random) sampling. Respondents from all throughout Ardabil city were sought for the research. The present researchers shared the survey link on social media (i.e., WhatsApp, Telegram channel) and via email with idividuals at the age of 20 and above (since, at the time of study, this age group were required to get the vaccine). The questionnaire for the study included sections on demographic and personal aspects connected to the COVID‐19 vaccine booster (annual boosters). A total of 662 people were interviewed for this study. Inclusion criteria were that adults over the age of 18 were eligible to receive two doses of the vaccine, 6 months had passed since the injection of the last dose, and they expressed a willingness to participate. Exclusion criteria were unwillingness to participate in the study, age under 18 years, and not receiving two doses of the vaccine.

### 2.2 Survey instrument

Information required for this study was measured by asking the question: “Would you like to get a booster dose if you have one?” on a 4‐point scale: 1 = *No*, *never*, 2 = *No*, *but maybe in the future* (*as soon as*), 3 = *I cannot decide*, 4 = *I am already vaccinated*.

### 2.3 Independent variables

In this study, independent variables were segments of socioeconomic, demographic, and personal variables. These factors included gender, age, level of education, occupation, healthcare professionals, marital status, and the purpose for rejecting or postponing the booster shot. These reasons included (uncertainty about the effectiveness of the booster shot, the presence of side effects after getting the vaccine, the belief that the doses of vaccines taken give sufficient immunity, and the belief that the booster dose is unnecessary (incorrect or contradictory information).

Both closed and open‐ended questions were used to collect sociodemographic information. In addition, individuals were questioned about their family's diagnosis of previous illnesses (diabetes, hyperlipidemia, lung disease, stroke, autoimmune disease, etc.).

Specialists in health education, psychiatry, and women's health checked that the survey was real and that it was true to its content. This was used to judge how reliable the questions were. The validity of the instrument was tested in two ways: face validity and content validity, as substantiated by health education experts. The total content validity index in “relevance” of content, “simplicity,” and “clarity,” was, respectively, 82.6, 92.9, and 90.7. The reliability of the instrument was tested further using the internal consistency test (*α* = 0.83) as well as the test‐retest reliability (*r* = 0.82).

### 2.4 Analysis of statistics

The analysis was performed using the SPSS software (v.23). The data was reported scientifically as the mean, the standard deviation, and the percentages. The Pearson correlation coefficient was utilized to investigate the existence of a connection between numerical variables, while chi‐square analysis was utilized to ascertain the nature of the link that existed between the ordinal variables that were being examined. Regression was also used for predictive factors. In each of the tests, a threshold of significance of *p* less than 0.05 was adopted.

### 2.5 Ethics approval and consent to participate

The present research was conducted in accordance with the Helsinki Declaration. The ethical standards for scientific research procedures were adhered to. The Ethics Committee of the Ardabil University Medical Sciences (#IR.ARUMS.REC.1401.126) confirmed this study. All participants were informed of the study, and just those who signed a written informed consent form were included in the study.

## 3 RESULTS

### 3.1 Demographic and baseline characteristics

There were a total of 662 people who responded to the survey. Among the 662 individuals who had been invited to take part in the research, 559 completed the questionnaire (response rate, 84.4% and 15% as the rate ofno response). In Table 1, you will see a breakdown of the research group's demographics and other relevant details. The majority of participants had at least a bachelor's degree (63.35 In Table 1, you will see a breakdown of the research group's demographics and other relevant details.

**Table 1:** Demographic features of participants (*n* = 662).

Variable	Group	*N* (%)/*M* ± *SD*
Age		39/02 ± 9/08
Sex	Female	351 (53/02%)
	Male	311 (46/9%)
Place of residence	Rural area	211 (31/87%)
	Urban area	451 (68.12%)
Level of education	Diploma and lower	236 (35/64%)
	College degree	426 (63/35%)
Marital status	Married	583 (88/06%)
	Single	79 (11/93%)
Healthcare workers	Yes	102 (15/4%)
	No	560 (55/33%)
Chronic conditions	Yes	509 (76/88%)
	No	153 (23/11%)
Side effect following vaccination	None	156 (23/56%)
	Mild	434 (65/55%)
	Severe	72 (10/87%)

A total of 11% of the population had at least one chronic ailment, but only 4% of the population worked in the healthcare industry. Most of the time, respondents were given the BBIBP‐CorV vaccine (74.8% of the time), followed by the AZD1222 vaccine (10.1% of the time). A total of 51.9% of the participants alluded to the emergence of adverse effects after the initial program, with the majority of the participants being minor (69.8%).

Attitudes toward the COVID‐19 vaccination booster dose: Among all the participants, 238 (35.9%) had previously gotten the COVID‐19 vaccine injection dose, and 198 (29.2%) prefered to obtain it ASAP. A total of 187 of the respondents, which is 28.2%, said that they were not going to take the booster dosage, and 39 of them, which is 5.8%, had not determined yet.

The most common reason given by 301 (45.4%) of those who responded to the survey for choosing not to receive the booster dose or delaying its administration was doubt about the booster dose's usefulness and its harmful effects. They were worried about the future effects of the vaccine on their health.

The second most common reason among participants was misinformation about booster shots. The information heard, without knowing if it was true, influenced the participants' decisions. Regression analysis showed the most common reasons for the reluctance to vaccinate were the uncertainty about the effectiveness of the shot dose, the side effects (odds ratio [OR] = 5.42, 95% CI = 4.1–6.7), and misinformation or contradictory information (OR = 4.33, 95% CI = 2.32–6.87). Moreover, those trusting the health system most likely were to get the vaccine (OR: 2.26; 95% CI: 1.01–1.56), and individuals with a higher perceived risk of getting infected were 3.83 times (OR: 3.83; 95% CI: 3.78–6.17) higher odds of receiving the vaccine.

Table 2 gives a full look at the different points of view on the booster dose and the reasons why it was rejected or put off.

**Table 2:** Eagerness to get the vaccination and explanations for refusal to be vaccinated or postponement of the booster shot.

Variable	Reasons	*N* (%)
Eagerness to receive the booster shot (COVID‐19)	No, not ever	187 (28.2%)
	No, but maybe in the future	198 (29.9%)
	I cannot choose	39 (5.8%)
	I am already vaccinated	238 (35.9%)
Reasons for not being interested in the booster shot or putting off giving the booster dose (COVID‐19) (*n* = 662)	Uncertainty about effectiveness of the booster dose and side effects	301 (45.4%)
	Event after taking the vaccine(trust in the health system)	41 (6.19%)
	My previous vaccinations have provided me with an adequate level of protection against infectious diseases. (perceived risk)	95 (14.3%)
	Relate of complications in friends and family	26 (3.9%)
	Issues have been raised over the vaccination and the potential adverse effects it may have in the henceforward. (misinformation or contradictory information)	199 (30%)

### 3.2 Effects of demographic variables and concerns on willingness to take the booster dose

According to the findings of an investigation into the effects of various demographic factors, the level of maturity of respondents' readiness to be vaccinated rose with age (*r* = 0.4 *p* = 0.01). %). In other words according the multivariate model, participants older than 40 years old (OR: 0.73; 95% CI: 0.52–0.94), were significantly associated with vaccine acceptance (*p* < 0.05).

Other results showed 64.7% of health personnel surveyed said that they had either already been immunized or wished to obtain the vaccine as soon as it was available. This indicates that healthcare experts are more inclined to get vaccinated. On the other hand, there was no correlation between vaccination views and factors such as gender, relationship status, standard of education, or current address. There is a significant correlation between the presence of many chronic diseases and an increased desire to get the COVID‐19 booster dose vaccination (*p* < 0.001) Also, *p* = 0.01), and following the news about COVID‐19 (*β* = 0.07, *p* = 0.01) were the predictors of subjects' attitudes toward the regular COVID‐19 vaccine.

## 4 DISCUSSION

Demands for receiving vaccines have waned since the COVID‐19 pandemic.^1^ With the prevalence of various novel variants instigated by the widespread circulation of the SARS‐CoV‐2 virus and the reduced level of protective antibody titers from previous vaccine dosages in populations, the vaccine shot appears to reduce the COVID‐19 pandemic and help achieve more excellent safety against the virus.^2^, ^3^ The purpose of this research was to evaluate the opinions of adults living in Ardabil, Iran, about the administration of COVID‐19 vaccination booster doses as well as the variables that are associated with this decision. In our study, approximately 35.9% of the participants reported they had taken a booster dose of the COVID‐19 vaccination, and about 28.2% of participants reported they did not want to take a booster dose. Concerns about side effects of the booster dose(s) and misinformation about vaccination were two factors for not being interested in receiving the booster shot(s). Age, suffering from chronic diseases, educational level, and gender were some demographic characteristics that can affect people's attitudes toward the intake of booster doses.

The study found that 35.9% of the participants had received vaccine boosters (annual boosters), 29.2% were willing to receive the dosages soon, and 28.2% stated they were unwilling to receive any shot doses. According to a study led in Italy, 85.7% of the participants specified that they were eager to get the booster shots.^4^ A study in Algeria suggested that 13.2% of the people had received COVID‐19 vaccine boosters (annual boosters), and 25% had refused any such doses.^5^ Another study showed that 67.4% of the Polish respondents had called for the timely reception of the boosters. Among the respondents, 2.5% had previously had the booster doses, while 2.9% did not plan to get immunized at any point in the future.^6^ This difference in response may have been due to the place and time of gathering data for studies and factors affecting the participants' attitudes,such as demographic differences, media coverage, and government policies to fight against the COVID‐19 virus. Other studies have reported different rates of people willing to receive booster doses; for example, 55.3% of healthcare workers in Saudi Arabia,^26^ 71% in Poland,^14^ 71.3% of healthcare workers in Czech,^15^ 79.1% of U.S. adults,^16^ 84.5% of Japanese medical students.^19^ In Omidvar and Firouzbakht study^20^ acceptance rate of the vaccine among Iranian participants was approximately 70%. The reception of vaccine booster (annual booster) doses in developed nations is generally higher. Maybe, the higher rates were due to positive experiences with previous doses and confidence in the efficacy of the vaccines.

Per the findings of our research, a mistrust of the efficiency of the booster dosage was perhaps the most effective factor in people's decisions not to have the COVID‐19 vaccine, and concerns about the occurrence of side effects were another important factor in this decision. Similar to this, other research found that the most prevalent reason given by people for their lack of desire to be vaccinated was a dearth of faith in the efficiency of booster shots and the difficulties that may arise from receiving them.^6^ In another study, fear of complications from the vaccine was the fourth reason for reluctance to receive booster doses.^5^ Thirty percent of individuals in the first 4 months of 2021 reported concern about possible complications. Therefore, they did not want to receive any vaccine against COVID‐19.^22^ Doubts about booster doses, followed by fear of complications, may be related to previous vaccine experiences. Some people also found that booster doses could end up causing more severe complications than previous doses.^3^ Another study found that 95.7% of 346 participants showed an increase in the titer of IgG antibodies on the tenth day after the vaccination booster.^23^ The conduct of more research and the release of more findings were associated with an increased level of people's knowledge and a reduced rate of scepticism over vaccination.^24^, ^27^

Misinformation about immunizations was the second most common reason why people didn't want to get their shots renewed. Misinformation was one of the concerns of participants for vaccine acceptance in Tehran.^28^ The widespread circulation of false stories on social media about the COVID‐19 vaccinations has contributed to the spread of misinformation.^29^ People with less money and more mental distress are more likely to have the wrong ideas about COVID‐19.^19^, ^29^ The World Health Organization has taken steps to combat disinformation and misinformation, one of which is optimizing web searches to locate reputable and authoritative resources with answers to concerns concerning COVID‐19^30^ In addition to the publishing of warnings and notifications, as well as links to reputable sources, while looking for information regarding vaccinations.^31^ Campaigns have been launched with the goals of providing accurate information and raising public awareness online, taking active actions against misinformation about the COVID‐19 immunization, and increasing the number of people who get booster doses.^28^ Although the efficacy of the vaccines in preventing the disease decreases over time,^21^ it should be stated that this efficacy will undoubtedly provide more excellent protection against more severe COVID‐19, hospitalization, and death.^32^, ^33^ Thus, to protect people and raise their safety against more severe complications of the diseases, policymakers are required to consider the prescription of extra boosters in the future.

Differences in demographic characteristics that affect people's attitudes, play a role in determining whether or not they are willing to take doses of the COVID‐19 vaccination.^22^, ^23^ We found that a higher readiness to accept booster dosages occurred with increasing age. In addition, it was shown that those who suffer from chronic ailments have a stronger resistance to taking booster dosages. Other researchers found that people of older ages^21^, ^22^, ^28^, ^34^ and those with chronic conditions were more willing to get the COVID‐19 vaccination dosages than the healthy ones. On the other hand, people with chronic illnesses have been shown to have results that don't fit with these findings.^34^ Older people had more enthusiasm to receive booster doses because of the underlying diseases, which made them develop a severe illness status and risk more fatalities from the viral infection. Thus, these people saw the injection of booster doses as an urgent need. Health officials should pay attention to these results. Prioritize the injection of extra booster doses. Also, they should provide the necessary training on the knowledge related to the COVID‐19 vaccines and plan for more action in this regard because their general improvement status is associated with receiving booster doses.

The result analysis of our study suggested that healthcare workers had a greater desire to be vaccinated with booster doses, with 64.7% of them expressing that they had already been vaccinated or sought to receive extra inoculation. Similarly, healthcare workers of various nations reported a higher acceptance of initial vaccines and shot vaccines for COVID‐19.^15^, ^35^, ^36^ During their routine tasks, healthcare workers are in direct contact with patients; on the one hand, they can be regarded as a source of explaining and removing public skepticism over vaccination and the dissemination of vaccination using primary medical data in the community. Therefore, removing skepticism over receiving vaccine booster (annual booster) doses in this group is highly important.

It has been found that higher education levels are directly related to receiving booster doses.^16^ Consistent with our findings, a higher percentage of respondents with academic degrees than those with high school or diploma degrees stated they were willing to have extra booster shots. However, this rate was not statistically significant. Another study showed that the more education, the more acceptance of booster shots.^5^ In another study, the vaccine acceptance rate was reduced in participants with higher levels of education.^20^

Similar to Jørgensen's^37^ study, the present study reported no difference in willingness to receive booster doses from a gender perspective. However, some other studies have concluded that females are more willing to receive booster shots. Other studies, conversely, demonstrated that men had a more significant incline toward the extra shots.^15^, ^16^, ^38^ There are two possible explanations for this difference. The COVID‐19 infection is nearly as serious an infectious illness for women's health as it is for men's. Women showed more adaptability to public health policies during COVID‐19 than men.^29^ Another possible explanation for the low acceptance rate of the booster dose among women is some misinformation circulating on social media about the COVID‐19 vaccination that has centered around defective female fertility or congenital disabilities.^30^, ^31^, ^39^ The use of training interventions about COVID‐19 vaccinations, benefits, and complications can increase the public's desire to inject extra shots. In light of people's changing perceptions of COVID‐19 and the increasing circulation of information about vaccinations, people's skepticism over shots could change over time.^34^ However, more actions should be taken to change people's attitudes and increase their acceptance of booster doses so that possible viral variants can be prevented. General health officials and responsible organizations can adopt policies, training, and strategies for all people, especially at‐risk groups, to expand the coverage of booster doses of COVID‐19.

### 4.1 Limitations

One limitation of the present research was the intermittent availability of the vaccine. occasionally, the vaccine was scarce, and there were times that it was abundant. Another limitation was the public mistrust culture, because some subjects experienced their loved ones' death. Simultaneously, they had been already vaccinated, and attributed this state to the undesirability of vaccines. As experienced in different waves of the pandemic, people were no longer as scared of the disease as they used to be; that is to say that through time they had lost their fear. Thus, the time spent carrying out this research can influence the findings, which was out of the present researchers' control.

## 5 CONCLUSION

According to the findings of the research, doubt about the booster dose's usefulness and its harmful effects, misinformation about booster shots, distrust of the health system, low risk perception as, are four important factors in not accepting the booster dose, on the other hand, herd immunity requires maximum vaccination. It is essential that ongoing monitoring of attitudes regarding primary immunization as well as shot dosages be performed, and that the general population be actively encouraged to get vaccinations. In addition to these identified barriers, it is necessary to improve people's attitudes and reduce the spread of false information among people, which is possible with the participation of people along with health system employees, People's participation, hearing ambiguities from people's language and clarifying is the most important factor of trust between people and the health system, which ultimately leads to improving the quality of life and improving the health of society.
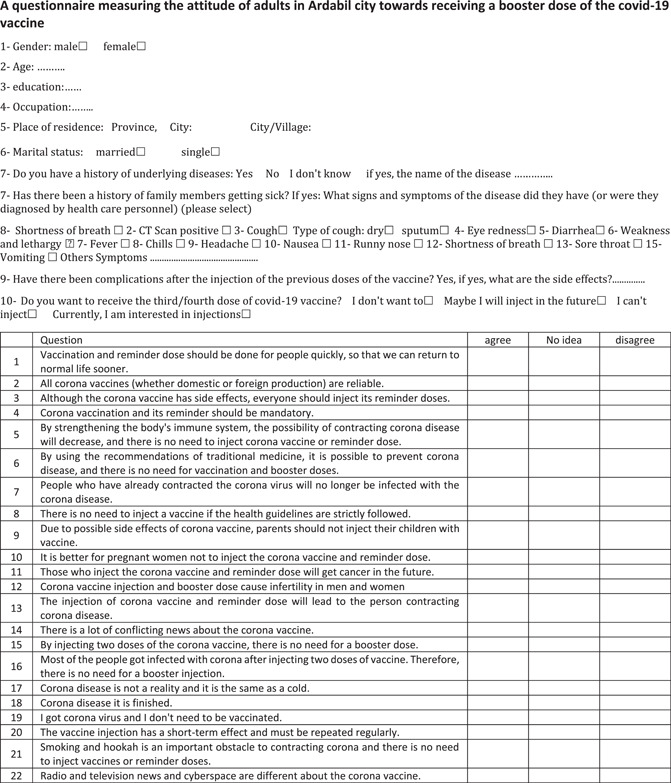


## AUTHOR CONTRIBUTIONS

**Nazila NeJhaddadgar**: Conceptualization; writing—original draft; writing—review and editing. **Mohammad Jafarzadeh**: Data curation; methodology; resources. **Zahra Khazir**: Data curation. **Javad Yoosefi Lebni**: Project administration; supervision; writing—review and editing. **Mohammad Rostami**: Data curation; writing—original draft. **Parisa Janjani**: Investigation; supervision; writing—review and editing. **Arash Ziapour**: Conceptualization; writing—original draft; writing—review and editing.

## CONFLICT OF INTEREST STATEMENT

The authors declare no conflicts of interest.

## ETHICS STATEMENT

The present research was conducted in accordance with the Helsinki Declaration. The ethical standards for scientific research procedures were adhered to. The Ethics Committee of the Ardabil University Medical Sciences (#IR.ARUMS.REC.1401.126) confirmed this study. All participants were informed of the study, and just those who signed a written informed consent form were included in the study. This consent was obtained as well from the authorities in charge—tacitly or explicitly—at the institute/organization where the research was conducted, before the work was submitted for publication. The purpose of study was comprehensively explained to the subjects in the cover page of the questionnaire. The subjects were assured of the confidentiality of the information they provided. An informed consent was obtained from all participants to show agreement to take part in the study having reviewed the cover page of the questionnaire and clicking on the associated link. Moreover, for subjects below 18 years of age, the subject was asked for the consent of the parent or his/her guardian.

## TRANSPARENCY STATEMENT

The lead author Arash Ziapour affirms that this manuscript is an honest, accurate, and transparent account of the study being reported; that no important aspects of the study have been omitted; and that any discrepancies from the study as planned (and, if relevant, registered) have been explained.

## Data Availability

The datasets using in the research can be available from the corresponding author on reasonable request.
